# No Effect of Dietary Aspartame or Stevia on Pancreatic Acinar Carcinoma Development, Growth, or Induced Mortality in a Murine Model

**DOI:** 10.3389/fonc.2017.00018

**Published:** 2017-02-09

**Authors:** James Dooley, Vasiliki Lagou, Tom Dresselaers, Katinka A. van Dongen, Uwe Himmelreich, Adrian Liston

**Affiliations:** ^1^Translational Immunology Laboratory, VIB, Leuven, Belgium; ^2^Department of Microbiology and Immunology, KU Leuven – University of Leuven, Leuven, Belgium; ^3^Biomedical MRI/MoSAIC, Department of Imaging and Pathology, KU Leuven – University of Leuven, Leuven, Belgium

**Keywords:** aspartame, stevia, pancreatic cancer, *in vivo*, MRI

## Abstract

Pancreatic cancer has an extremely poor prognosis, largely due to a poor record for early detection. Known risk factors for pancreatic cancer include obesity, diet, and diabetes, implicating glucose consumption and regulation as a key player. The role of artificial sweeteners may therefore be pertinent to disease kinetics. The oncogenic impact of artificial sweeteners is a highly controversial area. Aspartame, one of the most studied food additives, is widely recognized as being generally safe, although there are still specific areas where research is incomplete due to study limitations. Stevia, by contrast, has been the subject of relatively few studies, and the potential health benefits are based on extrapolation rather than direct testing. Here, we used longitudinal tracking of pancreatic acinar carcinoma development, growth, and lethality in a sensitized mouse model. Despite exposure to aspartame and stevia from the *in utero* stage onward, we found no disease modification activity, in either direction. These results contribute to the data on aspartame and stevia safety, while also reducing confidence in several of the purported health benefits.

## Introduction

Pancreatic cancer has one of the highest fatality rates, making it the fourth highest killer in absolute numbers despite being relative rare in incidence (1). A major contributor to high mortality is late detection—more than half of pancreatic cancers are diagnosed at a late stage, where 5-year survival rates are only 2% (1). This makes identifying the risk factors for pancreatic cancer imperative. Currently, age, smoking, obesity, lack of physical activity, diet, type 2 diabetes (T2D), chronic pancreatitis, cirrhosis, and genetic background are all associated with pancreatic cancer risk (2, 3). The association of obesity, diet, and T2D with pancreatic cancer illustrates the strong linkage to glucose intake and regulation, thereby identifying artificial sweeteners as an important topic for investigation.

Aspartame is the most commonly used artificial sweetener. The modified dipeptide of aspartic acid and phenylalanine gains it property of sweetness through binding TAS1R2 and TAS1R3 (4). The use of aspartame as a food additive originally faced widespread resistance due to carcinogenic fears, with increased risk of cancer in some studies of treated rodents (5, 6), although the study design has been criticized (7). Epidemiological analysis of aspartame use identified additional risk of lymphoma (8), urinary track tumors (9), and prostate cancer (10). However, this risk identification has been small and inconsistent, and independent studies have not reproduced the same result (11–13). Overall, thorough review of the scientific literature has found no robust and convincing evidence for toxic effects, including cancer promotion (14–16). Nonetheless, aspartame remains controversial (17, 18), especially in the public sphere, with prominent media attention given to several anti-aspartame campaigners.

While much of the criticism of aspartame seems scientifically unwarranted, there are gaps that merit further research. Epidemiological studies on aspartame use will always struggle to pick up weak disease modification associations, as studies tend to rely on self-reported consumption of aspartame-sweetened (diet) beverages as the proxy for aspartame exposure, while aspartame is actually added to more than 6,000 consumer products. Self-reported diet drink consumption is also not randomly assigned and will show correlation with other variables that may modify disease. Consumption also changes over time, with past consumption much more difficult to assess, so impacts of long-term aspartame use will be harder to identify. Likewise in animal studies, toxicity studies are well designed to pick up strong disease induction effects, but since they start with a healthy population of animals they are unlikely to identify disease modification effects. For a rare disease such as pancreatic cancer, a risk modification of even 10-fold increase is thus unlikely to be picked up in most studies, driving the need for screening the dietary impact in sensitized animal models.

In contrast to aspartame, stevia is widely cited in alternative medicine circles to have antitumor properties, albeit with little scientific foundation. Stevia is an artificial sweetener extracted from the leaves of *Stevia rebaudiana*, the sweetness of which can be attributed to steviol glycosides (mainly stevioside and rebaudioside) (19). Stevia and its derivatives were originally banned in some countries due to fear of potential carcinogenic properties; however, toxicology studies found physiological doses to be safe (20). The origin for the claim of antitumor properties can be traced back to studies of skin cancer formation in mice. Using the model of 12-*O*-tetradecanoylphorbol-13-acetate and dimethylbenz[a]anthracene skin exposure, the topical application of stevioside mixture reduced the incidence of skin tumor formation (21, 22). While this may reflect antitumor properties, the experimental result could equally represent an anti-inflammatory or even a skin barrier enhancement function. Regardless, the potential topical properties of stevia cannot be extrapolated out to systemic effects after dietary intake. To our knowledge, no studies on dietary stevia supplementation have been performed on cancer, leaving a critical gap in our knowledge which has been filled by inflated claims from alternative medicine practitioners.

Here, we have sought to determine the impact, if any, of aspartame and stevia on pancreatic acinar carcinoma. With strong links to diabetes, glucose consumption and obesity, pancreatic cancer remains one of the strongest candidates to detect an oncogenic role, either promoting or suppressive, of artificial sweeteners. Despite the widespread safety studies of aspartame, the effect on pancreatic cancer remains opaque, as the rareness of the disease would preclude detection of risk modification in epidemiological studies. In the case of stevia, the near-complete absence of solid experimental data for dietary supplementation in cancer makes *in vivo* testing imperative. Through dietary supplementation and longitudinal magnetic resonance imaging (MRI), we find that neither aspartame nor stevia have any significant impact on pancreatic acinar carcinoma development, growth, or mortality.

## Materials and Methods

### Mice

C57BL/6 Ela1-TAg mice were purchased from Jackson (23, 24). Mice were bred under specific pathogen-free conditions and from the time of breeder set-up were exclusively fed on a standard chow (ssniff^®^ R/M-H) with either normal drinking water, or drinking water supplemented with either aspartame (0.035% w/v, Blackburn Distributions Ltd.) or stevia (0.02% w/v, Stevia Natura) *ad libitum*. Male mice were moved to conventional conditions at 7 weeks of age for longitudinal MRI, with continuation of the dietary restriction. The study and the protocol were approved by the University of Leuven Animal Ethics Committee. All experimental procedures were carried out in accordance with the recommendations of the University of Leuven Animal Ethics Committee. Mouse-weight and blood glucose were monitored throughout the experimental time course. Mortality indicates the experimental end-point, defined as the loss-in-condition sufficient to trigger ethical euthanasia (by carbon dioxide).

### Imaging

A Bruker Biospin 9.4 T Biospec small animal MR scanner (Bruker Biospin, Ettlingen, Germany) was used to image mice under isoflurane anesthesia. The scanner was equipped with an actively shielded gradient set of 600 mT/m using a respiration triggered spin echo sequence with 50 continuous slices of 0.5 mm thickness in interlaced mode (acquisition parameters: repetition time = 6,000 ms, echo time = 15.9 ms, field of view = 4.0 cm × 6.0 cm, a matrix of 200×400, two dummy scans and two averages). For radio-frequency irradiation and detection, a 7.2 cm quadrature resonator was used.

### Data and Statistical Analysis

Images generated by MRI were analyzed with ImageJ to identify tumors (National Institute of Health, Bethesda, USA). Each individual tumor was assessed in cross-section for the maximum tumor radius, which was used to predict volume using the formula: $4/3\times \mathrm{area}\times \sqrt{\left(\mathrm{area}/\pi \right)}$. Statistical analysis was performed in R (https://www.r-project.org/ version 3.1.2). Cumulative incidence curves were generated using the R package “survplot” with fun = function(*x*) {1 − *x*} (25). Survival curves were generated using the R “survplot” package and the Kaplan–Meier log-rank test, implemented in the R “survdiff” package (26).

The linear mixed-effect model included the cross level interaction between time and diet (i.e., the effect of time is allowed to vary between diet groups). We also considered time to have variable effects to allow for the change in tumor growth over time to differ across participants (i.e., explicitly model individual differences in change over time). The formula of the model is as follows: tumor growth ~ time + diet + time × diet + (1 + time|subject). This model provides a fixed-effect estimate for the interaction between change over time and diet that indicates whether the rate of change with respect to tumor growth is significantly different between the regular water and other diets. We considered the regular water diet as reference diet and week 7 as reference time. These linear mixed-effect models were fitted within each sex using the lmer function within lme4 (Linear mixed-effects models using “Eigen” and S4) package in R.

## Results

### No Impact of Dietary Aspartame or Stevia on Pancreatic Acinar Carcinoma Kinetics

**Figure 1:**
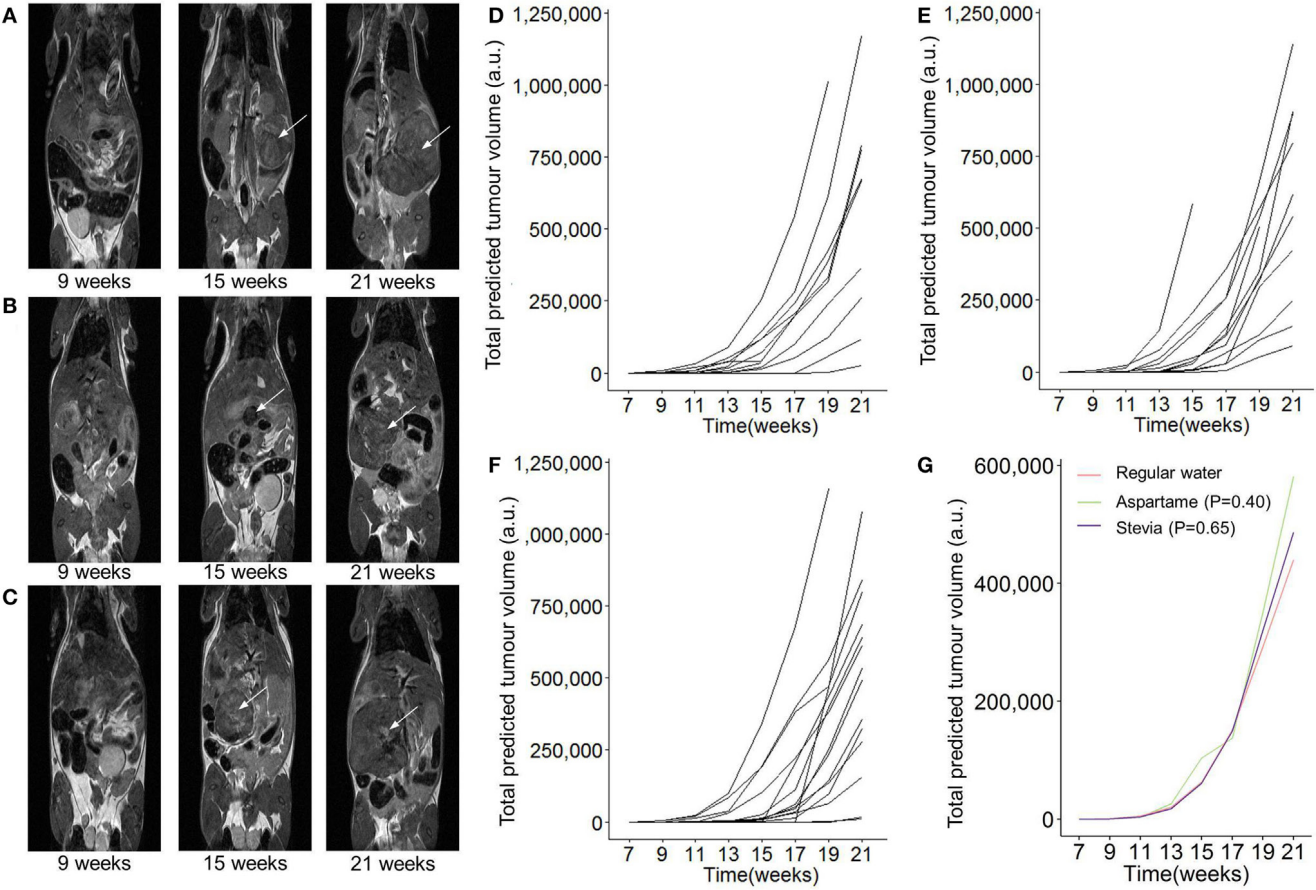
**Longitudinal monitoring of pancreatic tumor growth following dietary addition of artificial sweeteners**. TAg^+^ mice were placed on alternative water supplies *in utero*, and aged on the same exposure to 21 weeks of age. From 7 weeks onward, mice were assessed through magnetic resonance imaging (MRI) for tumor detection and size. Representative MRI tumor images for mice with **(A)** normal drinking water, **(B)** drinking water supplemented with aspartame, **(C)** or drinking water supplemented with stevia at 8, 14, and 20 weeks. **(D)** Individual total predicted tumor volume curves for mice with normal drinking water (*n* = 13), **(E)** drinking water supplemented with aspartame (*n* = 12), **(F)** or drinking water supplemented with stevia (*n* = 15). **(G)** Grouped total predicted tumor volume curves for the data in panel **(D–F)**. *P* values indicate effect of treatment vs normal drinking water, using a linear mixed-effect model. Time corresponds to the age of the mouse.

**Figure 2:**
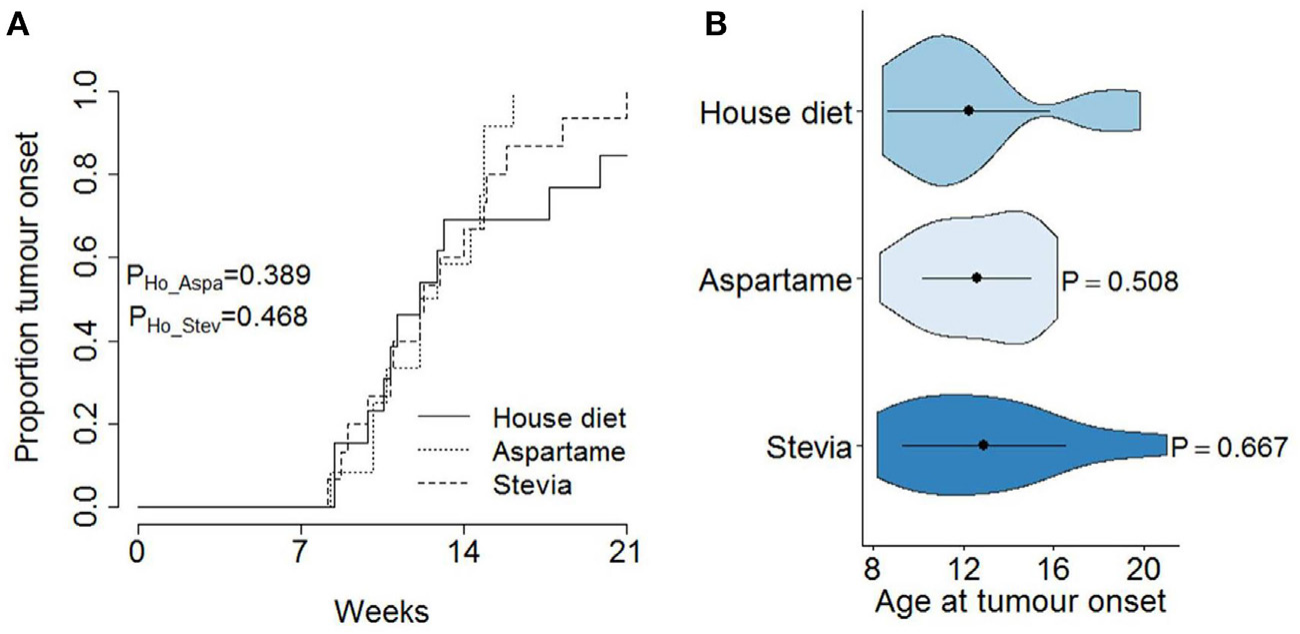
**No effect of artificial sweetener on pancreatic cancer development**. TAg^+^ mice were placed on alternative water supplies *in utero*, and aged on the same exposure to 21 weeks of age. From 7 weeks onward, mice were assessed through magnetic resonance imaging for tumor detection. **(A)** Cumulative incidence of pancreatic cancer as a function of age at tumor onset, stratified by water supply (standard, aspartame, stevia) in male mice (*n* = 13, 12, 15). The *P* values were calculated using the log-rank test. **(B)** Violin plots showing the mean, SD, and kernel probability density of the age at tumor onset under each dietary modification. The *P* values were calculated using two-tailed unpaired *t* test.

Systematic *in vivo* testing of the potential impact of aspartame and stevia on pancreatic acinar carcinoma is required to identify any potential health gains or risks. We used the well-characterized Ela-TAg transgenic mice, which express the SV40 large T Antigen under the control of the Elastase-1 acinar cell promoter, driving spontaneous pancreatic cancer formation, of acinar origin (23, 24). This strain, while developing a less common form of pancreatic cancer, was chosen for the predictable tumor development course, allowing experimental testing of the impact of exposure while using relatively few mice. To ensure high exposure levels, TAg^+^ mice were placed on 0.035% w/v aspartame or 0.02% w/v stevia in the drinking water, without access to normal water. This dietary change was implemented at the point of breeder set-up, ensuring exposure at the *in utero*, neonatal, juvenile, and adult stage. From 7 weeks of age, TAg^+^ mice on either regular drinking water, drinking water supplemented with aspartame, or drinking water supplemented with stevia underwent MRI scanning every 2 weeks (Figures 1A–C). Following tumor detection, the volume of each tumor was calculated over the entire observation period, demonstrating exponential growth (Figures 1D–G). Based on the MRI data, the time of first tumor detection was calculated for each mouse. No effect of either aspartame or stevia was identified for the cumulative incidence of pancreatic acinar carcinoma (Figure 2A) or the age of tumor onset (Figure 2B). These results indicate that even with lifelong exposure, high doses of supplementary aspartame or stevia do not alter pancreatic acinar carcinoma development, with either a positive or negative effect.

**Figure 3:**
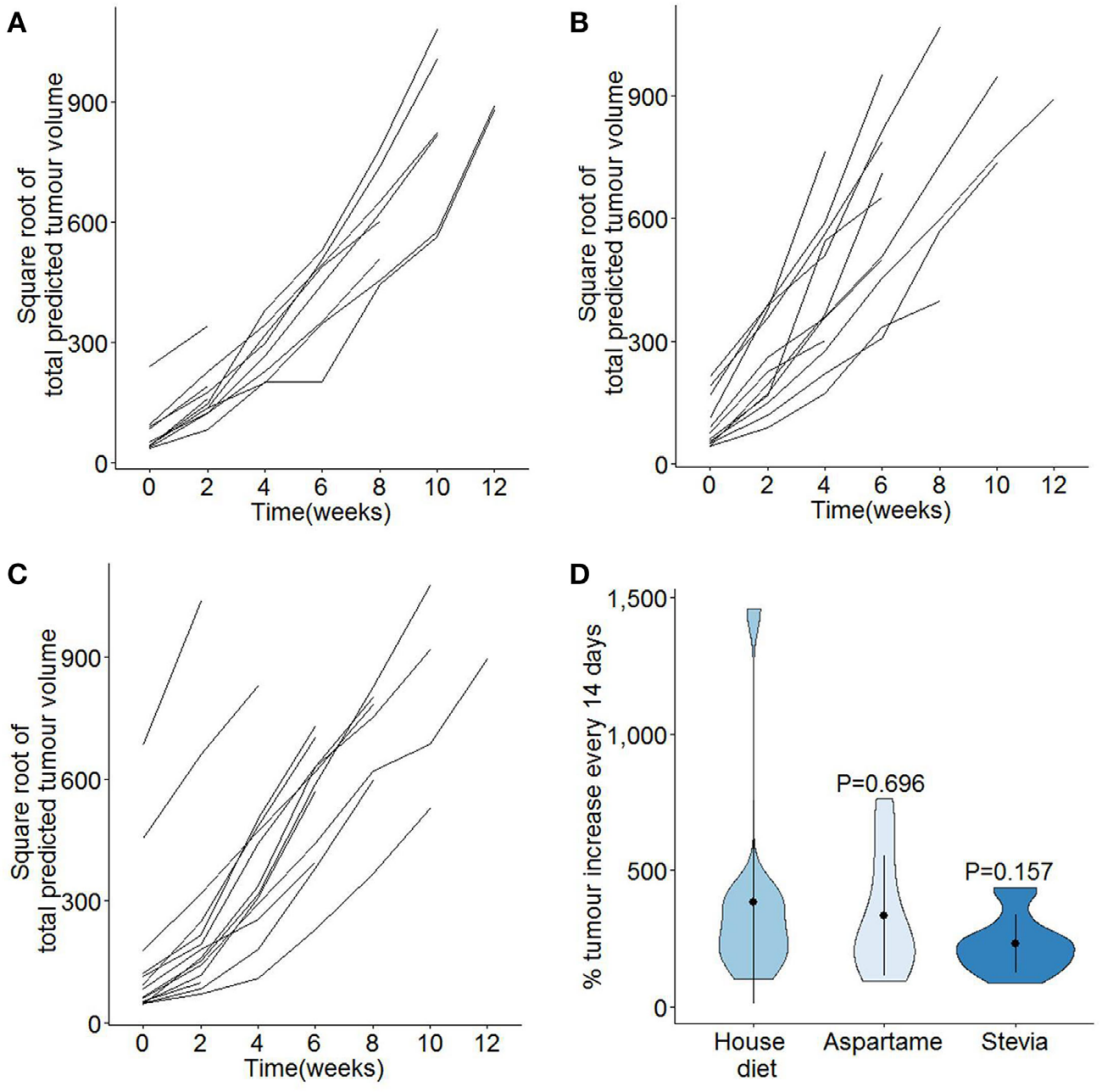
**Additional of high level dietary aspartame or stevia does not modify the growth rates of pancreatic cancer**. TAg^+^ mice were placed on alternative water supplies *in utero*, and aged on the same exposure to 21 weeks of age. From 7 weeks onward, mice were assessed through magnetic resonance imaging for tumor size. Individual square root transformed total predicted tumor volume curves for male mice on **(A)** standard drinking water (*n* = 13), **(B)** drinking water supplemented with aspartame (*n* = 12), and **(C)** drinking water supplemented with stevia (*n* = 15). Time 0 corresponds to the first detected tumor time-point. **(D)** Violin plots showing the mean, SD, and kernel probability density of the percentage of tumor volume increase every 2 weeks, averaged over the period of observation, under each condition in male mice. *P* values were calculated using two-tailed unpaired *t* test.

**Figure 4:**
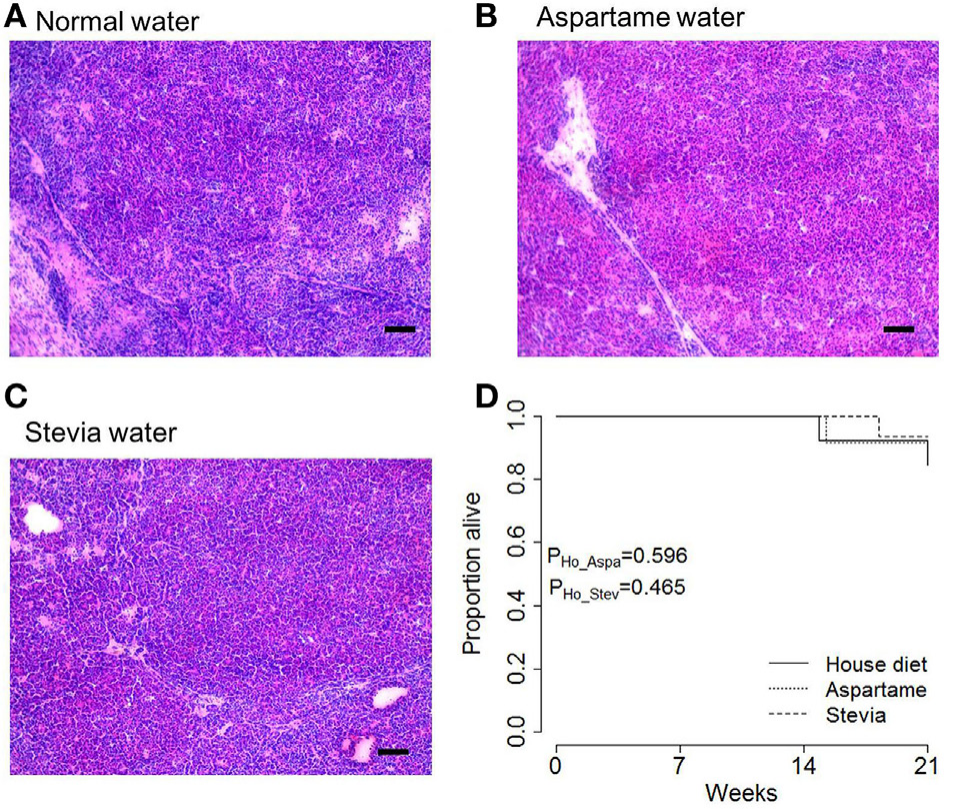
**Artificial sweeteners do not increase mortality rates in mice with pancreatic cancer**. TAg^+^ mice were placed on alternative water supplies *in utero*, and aged on the same exposure to 21 weeks of age. Mice were removed from the study when excessive morbidity was observed. **(A)** Representative H&E tumor histology from tumors extracted from male mice on standard drinking water, **(B)** drinking water supplemented with aspartame, and **(C)** drinking water supplemented with stevia, at 21 weeks of age. Scale = 100 µm. **(D)** Kaplan–Meier plot showing the overall pancreatic cancer survival in male mice on house, aspartame, and stevia diets (*n* = 13, 12, 15). *P* values were calculated using the log-rank test.

Following the assessment of pancreatic acinar carcinoma development, the impact of aspartame or stevia supplementation on the tumor growth rate was assessed. Total predicted tumor volumes were normalized for variation in age of onset and for the exponential growth rate (Figures 3A–C). This allowed the calculation of the tumor growth rate, defined as the average percentage of tumor volume increase per 2 weeks, for the period from tumor detection to experimental end (Figure 3D). By this measure, no alteration in the tumor growth rate was observed in mice given either the aspartame or stevia supplement. Finally, the effect of both artificial sweeteners on tumor histopathology and tumor-induced mortality was assessed. No systematic changes in histolopathology were observed (Figures 4A–C), and no increase in tumor-induced mortality over standard drinking water was observed (Figure 4D). Most TAg^+^ mice on standard drinking water survived for the entire observation period, limiting the ability to detect decreases in tumor-induced mortality; however, together these results argue against any profound effect of either aspartame or stevia on pancreatic acinar carcinoma growth or mortality induction.

## Discussion

Aspartame is one of the most tested of all food additives. The compound is rapidly metabolized into the constituent components (common amino acids) and is not found in the blood or urine of individuals that ingest it (27). This makes it unlikely to have any major carcinogenic effect, and, indeed, a meta-analysis of rodent testing indicates its safety (15). Despite this, the public concern over aspartame, divorced from scientific basis, is sufficient that major food companies have removed the artificial sweetener from selected products and replaced with alternative compounds (which, in the case of stevia, are less well studied than aspartame itself) (28). While this reaction is not proportional to the data, there are areas where more research is legitimately required. Pancreatic cancer is one of these areas, owing to the link with dietary glucose consumption and regulation. Our study demonstrated no detectable impact of dietary aspartame with pancreatic acinar carcinoma risk, using a well-defined sensitized rodent model.

Our results here also demonstrated no impact of dietary stevia on the kinetics of pancreatic acinar carcinoma. This is seemingly in contradiction with *in vitro* data from several laboratories, which demonstrate that various *S. rebaudiana* extracts have antiproliferative and proapoptotic properties on cancer cell lines (29–31), including on the pancreatic cancer cell lines MiaPaCa-2 (32) and BxPC-3 (33). However, when extrapolating to the physiological context, the process of digestion needs to be taken into account. For example, stevioside induces apoptosis in cancer cell lines *in vitro* (34), but a study of the human intestinal metabolism of stevioside found rapid hydrolyzation to steviol (35), and thus any potential anti-oncogenic properties of stevioside is unlikely to be felt outside of the gut. It is therefore likely that the pharmacological properties of studied *S. rebaudiana* extracts demonstrate a lack of physiological efficiency due to the exceeding local concentrations that would be generated.

Finally, it is worth noting that our study only discounts a direct role for dietary aspartame and stevia in modulating pancreatic acinar carcinoma, which in turn is a rare subtype of total pancreatic cancers (although with a high total mortality burden). Use of the precautionary principle may still advocate a limitation on the use of artificial sweeteners, even with negative data (17, 36). In our opinion, however, the precautionary principle in this case comes into direct conflict with a harm minimization approach. As artificial sweeteners, the main utility of both aspartame and stevia is as a substitute for sugar (37). Patient data suggests that consumption of sugar-sweetened beverages increases the risk of pancreatic cancer (38). Thus, regardless of the (lack of) direct bioreactivity of aspartame and stevia, when these compounds are used as a substitute for glucose, rather than an additive, they are likely to strongly reduce the risk of pancreatic cancer. The most pertinent question for the health impact of artificial sweeteners may there be the degree to which they substitute for sugar, a question perhaps even more controversial than the carcinogenic risk (39).

## Author Contributions

JD, KD, and TD collected the data. VL performed statistical analysis. UH and AL directed the study. JD and AL designed the study. AL wrote the manuscript, which was approved by all authors.

## Conflict of Interest Statement

The authors declare that the research was conducted in the absence of any commercial or financial relationships that could be construed as a potential conflict of interest.
